# Evaluation of a New Closed-System Automated RT-qPCR Assay for the Rapid Detection and Monitoring of Common Nucleophosmin Mutations in Patients with Acute Myeloid Leukemia

**DOI:** 10.3390/ijms25147912

**Published:** 2024-07-19

**Authors:** Richard D. Press, Dana Dressel, Michelle McBean, Ing S. Tiong, Matthew W. Anderson, David Pride, Aarthi Raman, Rachelle R. Doom, Rajesh Kaldate

**Affiliations:** 1 Department of Pathology, Knight Cancer Institute, Oregon Health and Science University, Portland, OR 97239, USA; 2 International Health Management Associates (IHMA), Schaumburg, IL 60173, USA; ddressel@ihma.com; 3 Peter MacCallum Cancer Centre, Melbourne, VIC 3052, Australia; michelle.mcbean@petermac.org (M.M.); ing-soo.tiong@petermac.org (I.S.T.); 4 Cenetron Diagnostics, Austin, TX 78758, USA; manderson@versiti.org; 5 Department of Pathology, University of California, San Diego, CA 92103, USA; dpride@health.ucsd.edu; 6 Cepheid, Sunnyvale, CA 94089, USA; aarthi.raman@cepheid.com (A.R.); rachelle.doom@cepheid.com (R.R.D.); rajesh.kaldate@cepheid.com (R.K.)

**Keywords:** acute myeloid leukemia, polymerase chain reaction, nucleophosmin (*NPM1*) mutation, monitoring, minimal residual disease (MRD)

## Abstract

Quantitative assessment of nucleophosmin 1 (*NPM1*) mutation status is integral to evaluating measurable residual disease (MRD) in *NPM1*-mutated acute myeloid leukemia (AML) patients. In a retrospective study, leftover peripheral blood (PB) specimens (n = 40) which were collected for routine clinical diagnostic evaluations of AML disease burden were tested by both a novel automated RT-qPCR quantitative NPM1 assay (Xpert NPM1 mutation assay) and the NPM1 mutA, mutB&D MutaQuant kit. Based on a Deming regression analysis, there was a high correlation (slope = 0.92; intercept = 0.12; Pearson’s r = 0.982) between the quantitative results of the Xpert NPM1 mutation assay and the NPM1 mutA, mutB&D MutaQuant kit. The Xpert test quantitative results are thus highly correlated with the comparator method and the former has potential as a useful alternative for the monitoring of AML patients with a known *NPM1* mutation.

## 1. Introduction

AML is a cancer of the hematopoietic stem cells in the bone marrow (BM) [1]. It is found primarily in adults over the age of 60 years, and in both men and women; it accounts for about 1% of all cancers. The yearly incidence of AML in the United States (US) and Europe is estimated at 4 per 100,000 members of the population. With 20,000 new cases of AML annually in the US and an estimated 50% or more of those cases resulting in death, AML is one of the leading causes of leukemia-related deaths [2]. A subset of AML patients are known to have various *NPM1* exon-12 insertion mutations.

The *NPM1* gene encodes a nucleolar phosphoprotein that regulates the transport of ribosomal particles between the nucleus and the cytoplasm, and is also involved in other cellular systems, including tumor suppressor pathways. *NPM1* is one of the most commonly mutated genes in AML, occurring in ~30% of all AML cases [3]. *NPM1* mutations were discovered following the observation that the protein abnormally localized to the cytoplasm in some AML patients. The subsequent genetic evaluation of leukemic blasts in patients with abnormally localized NPM1 led to the discovery of exon 12 (NM_001355006.1) frameshift mutations [4]. The most frequent *NPM1* mutations are the type A (~75–80%), type B (~10%), and type D (~5%); all of the nucleotide insertions in exon 12 result in a frameshift mutation, creating an aberrant C-terminal nuclear export signal (NES) which leads to the pathologic cytoplasmic localization of NPM1 and NPM1-interacting proteins. 

The National Comprehensive Cancer Network (NCCN) Guidelines recommend assessment of the *NPM1* mutation status in AML patients to aid in prognostication [5]. In patients with an *NPM1* mutation, persistence of the mutation after treatment can serve as a reliable marker of measurable residual disease (MRD), and can be monitored in the peripheral blood using real-time quantitative reverse transcription polymerase chain reaction (qPCR) [6]. The analytical sensitivity levels of *NPM1* single-gene targeted PCR-based tests are superior to levels associated with detection by most conventional next-generation sequencing (NGS) assays, and the former technique is considered the state-of-the-art technology for disease monitoring. The serial longitudinal monitoring of the *NPM1* mutation status during the patient’s entire post-treatment disease course is important because frank relapse is often preceded (by 2-3 months) by the detection of low-level MRD by a number of different laboratory methods [6]. 

We reasoned that both hematology–oncology clinical centers and molecular laboratories could benefit from a rapid, accurate, technically simple quantitative *NPM1* mutation assay to monitor their AML patients. Towards this goal, we present the results of the performance evaluation of a novel automated real-time qPCR cartridge-based *NPM1* mutation assay (Xpert NPM1 mutation assay) for the quantification of mutant *NPM1* mRNA transcripts (types A, B, and D in exon 12) using peripheral blood samples from *NPM1*-mutated AML patients. The Xpert NPM1 mutation assay was compared to the NPM1 mutA, mutB&D MutaQuant kit for each *NPM1* mutation subtype A, B, and D (the latter subsequently referred to as the Ipsogen assay). 

## 2. Results

A total of 40 PB specimens were obtained from 11 male (27.5%) and 29 female (72.5%) *NPM1*-mutated AML patients with an average age of ~50 years. Four specimens were excluded from the analysis because they were negative on the Xpert NPM1 mutation assay and/or the Ipsogen assay, together with sufficient *ABL1* transcript. Three of these specimens had undergone dilutions of 1:1000 (#39, #40) or 1:100 (#20), which lowered the percentage ratio to below the assays’ detection limit. Additionally, one specimen (#9) had undergone a dilution of 1:1 and was not detected by either method. A fifth specimen (#10) was excluded from the analysis because it was an outlier, having met the Grubb’s test criteria (G = 5.518 and *p*-value < 2.2 × 10^−16^), and thereby satisfied the requirements for an outlier. This outlier was possibly the result of an operator error, specimen handling issue, or a dilution process workflow error. Of the 40 *NPM1*-mutant AML patient specimens that were enrolled in the clinical study, 35 were within the quantitative ranges of both tests and were included in the analysis presented in Table 1. In agreement with other larger AML studies, the vast majority (77%) of NPM1 insertions were of subtype A, with only five recorded type-B and three type-D insertions [7]. To control for the quality and quantity of the RNA derived from the archived clinical lysates, the reference gene (*ABL1)* Ct values of all 35 samples (with the Ipsogen assay) were indicative of uniformly intact RNA (mean 24.4; range 22.8–25.6; CV 2.2%). The Xpert NPM1 mutation assay performed on exactly the same samples does not yield similarly informative ABL1 Ct data, as it measures the NPM1 to ABL1 transcript ratio using a relative (not absolute) expression calculation that includes a nested PCR method.

**Figure 1:**
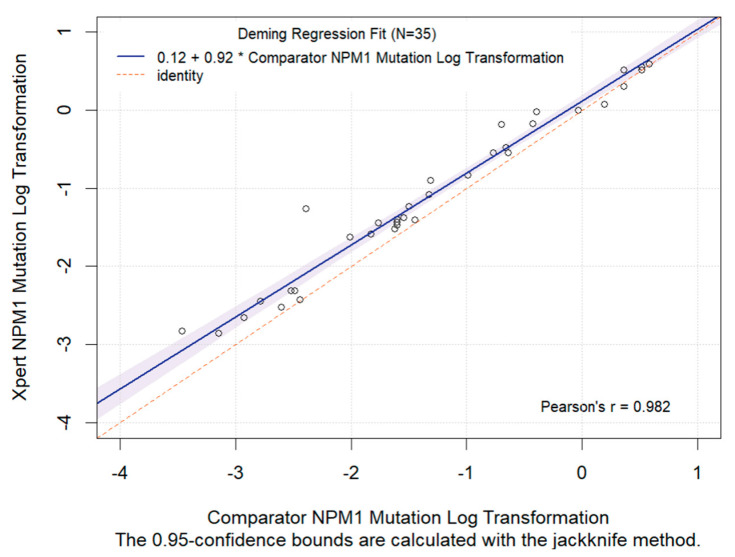
Deming regression analysis of log-transformed quantitative results (NPM1 RNA divided by ABL1 RNA) from the Xpert NPM1 mutation test and the comparator NPM1 mutation test, using lysates derived from peripheral blood specimens. The slope, intercept, Pearson’s correlation coefficient of the LT quantitative results and the regression line (solid line) are presented. Note that the shaded blue region is the 95% CI and the red dotted line is the identity line (X = Y).

Figure 1 below presents the Deming regression analysis including the slope, intercept, and line of identity of the log-transformed (LT) results. The 95% confidence interval (calculated using the jackknife method) and the Pearson’s correlation coefficient are displayed in the figure.

Figure 1 shows a high correlation (Pearson’s r = 0.982) between the LT results of the Xpert NPM1 mutation assay and the Ipsogen assay. The Deming regression analysis for LT had a slope of 0.92 and an intercept of 0.12.

**Figure 2:**
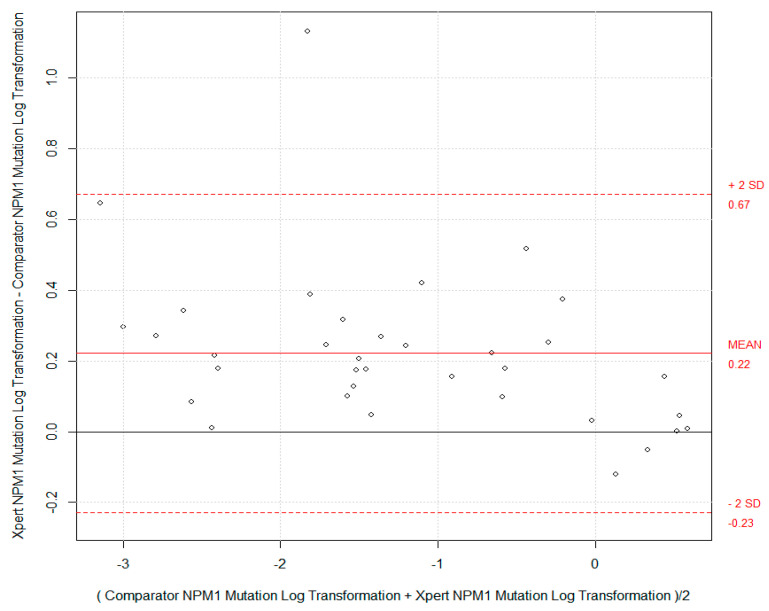
Bland-Altman bias plots showing the paired differences of the Xpert and Comparator test quantitative results on the y-axis versus the average of the pairs on the x-axis. The inter-test measurement variance was evaluated based on two standard deviations of the difference (dotted lines). The mean difference (solid red line) and the zero difference (solid black line) are presented.

A Bland–Altman plot was executed to evaluate the bias between the Xpert NPM1 mutation assay and the comparator method results for the 35 specimens included in the analysis, as presented in Figure 2. The Bland–Altman plot (Figure 2) showed that the Xpert NPM1 mutation assay yielded *NPM1* transcript ratios that were significantly higher than those from the comparator RT-PCR method (by 0.22 log; paired t-test *p*-value < 0.001). Despite this statistically significant bias, all but one specimen was within the plus/minus 2 SD (0.5 log) difference which is considered an acceptable level of inter-test measurement variance [8]. Overall, these results showed a high degree of correlation between the cartridge-based Xpert NPM1 mutation assay and the Ipsogen assay. 

Figure 2 shows Bland–Altman bias plots with the paired differences of the Xpert and comparator tests’ quantitative results on the y-axis versus the average of the paired measurements on the x-axis. The inter-test measurement variance was evaluated based on two standard deviations of the difference (dotted lines). The mean difference (solid red line) and the zero difference (solid black line) are also presented.

## 3. Discussion

In this study, the novel automated RT-PCR quantitative Xpert *NPM1* mutation assay and the comparator method (Ipsogen assay) yielded highly correlated quantitative results (albeit with a significant, but quantitatively minimal, measurement bias) across the entire dynamic range of the assays.The Xpert *NPM1* mutation assay showed a high level of agreement with the comparator method using whole-blood EDTA lysate samples spiked with MutA, MutB, and MutD *NPM1*-positive cell-lysates. In addition, and of considerable relevance to practical clinical decision-making, the total time to result (TTR) for the Xpert NPM1 mutation assay was under three hours, while the comparator assay had a TTR of approximately six hours, in addition to the time required for RNA extraction. Other possible advantages of the automated closed-tube system (relative to the comparator assay) include a potential reduction in PCR cross-contamination risk; the elimination of a separate manual RNA extraction step; and its applicability in patients with any of the three common NPM1 mutation types (A, B, or D), without pre-existing knowledge as to the exact insertion sequence. Possible disadvantages, however, include the absence of a purified leftover RNA preparation for additional research or clinical testing.

Limitations of this study include its retrospective nature (with stored archival samples), its inability to determine or distinguish the exact sequence of the NPM1 insertion (with the Xpert assay), and the absence of any direct clinical utility data correlating quantitative NPM1 transcript levels with clinical outcomes. This study was also intentionally not designed to address the important issue of defining and comparing the qualitative low-level detection limit of the NPM1 MRD target, which is, of course, a critical assay parameter for practical post-treatment disease monitoring (MRD) and prognostication.

The diagnosis and management of *NPM1* mutation-positive AML is commonly based on European Leukemia Net (ELN) and National Comprehensive Cancer Network (NCCN) guidelines [5,9]. The NCCN guidelines discuss the use of NPM1 mutations for MRD monitoring in *NPM1*-mutated AML patients. *NPM1* mutations are also prognostic in AML patients and are used to guide treatment decisions. These guidelines recommend that all AML patients be tested for *NPM1* mutations with RT-qPCR [5]. Next-generation sequencing (NGS)-based assays can be also used to detect *NPM1* mutations, but are not routinely used for MRD monitoring. In addition, the analytical sensitivity levels of PCR-based assays are superior to those associated with conventional NGS approaches [10]. The automated RT-PCR quantitative Xpert *NPM1* mutation assay described herein thus has the potential to offer physicians, labs, and their patients a convenient tool for the accurate, timely management of *NPM1*-mutated AML patients. 

## 4. Materials and Methods

### 4.1. Peripheral Blood Specimens and Clinical Lysates in Clinical Settings

Leftover blood specimens from forty (40) *NPM1* mutation-positive AML patients were collected under IRB approval as part of standard-of-care routine AML disease monitoring evaluations at Oregon Health and Science University (OHSU), Portland, Oregon. Those blood specimens that were *NPM1* mutation-positive and expressed Mut A, Mut B, or Mut D were processed into archived leukocyte lysates using a guanidinium thiocyanate-based lysis buffer (at 10 million cells per ml) and stored at ≤−80 °C (archived clinical lysates). These archived clinical lysate samples were variably diluted into cell lysates prepared from EDTA whole blood (WB) from healthy donors who had neither AML nor any evidence of *NPM1* mutations, and processed to final lysates prior to testing using the Xpert NPM1 mutation assay. 

The clinical lysates underwent a serial dilution to target the full dynamic range (0.03–500%) of *NPM1/ABL1* RNA levels. The 40 *NPM1* mutation positive final lysates in the study were diluted at 1:1, 1:10, 1:100 or 1:1000 dilutions (10 specimens at each level). The final lysates containing *NPM1* mutant RNA were quantitated using both the Xpert NPM1 assay and the RT-PCR quantitative comparator method (Ipsogen assay).

### 4.2. NPM1 Mutation Quantitation on the GeneXpert Instrument System

The Xpert NPM1 mutation assay (Cepheid) was performed on the Cepheid GeneXpert^®^ Instrument System. The GeneXpert Instrument System automates and integrates RNA extraction, reverse transcription (RT), nucleic acid amplification, and quantitative target sequence detection using real-time polymerase chain reaction (qPCR). The system consists of an instrument, a computer, and preloaded software (version 6.2 or higher) designed for running tests and viewing the results. The system requires single-use, disposable GeneXpert cartridges that hold the reagents for, and host, the real-time PCR processes. Cartridge-based testing was performed on the final clinical lysates and quantitatively interpreted by the system software (version 6.2 or higher) from measured mutant *NPM1* RNA relative to reference gene *ABL1* RNA using a delta Ct quantitation method (ΔCt = Ct_ABL_ − Ct_NPM1Mutation_) to determine the percentage ratio of *NPM1* mutant RNA divided by *ABL1* RNA. The assay’s limit of detection is 0.03% [11]. The assay’s detection limit and linearity are not significantly different for the 3 common NPM1 insertion types (A, B, and D) [11].

### 4.3. NPM1 Mutation Quantitation by Manual RT-qPCR following RNA Extraction 

RNA was extracted from the final clinical lysates using the Zymo Direct-zol™ RNA Miniprep Plus Kit (Zymo Research, Irvine, CA, USA). The RNA was quantified using the Thermo Scientific NanoDrop™ 2000 Spectrophotometer (Waltham, MA, USA) before being tested by the comparator test according to the manufacturer’s instructions for use, with slight modifications. RT was performed using the SuperScript™ VILO™ cDNA Synthesis kit (Thermo Fisher, Waltham, MA, USA) with 1500 ng RNA input, after which 200 ng of RNA was subjected to qPCR of *NPM1* mutant RNA relative to reference gene *ABL1* RNA, using the Ipsogen assay. The kit includes allele-specific PCR primers for *NPM1* mutations A, B, and D to quantitate *NPM1* mutations in RNA using a separate PCR reaction for each mutation. Co-amplified plasmid calibrators of a known copy number are used to quantitate each of the 3 transcripts. The test results are expressed as a ratio of *NPM1* mutant RNA relative to *ABL1* RNA. The assay’s limit of detection is 10 plasmid molecules, or approximately 0.1% [12].

## 5. Statistical Analysis

Samples with an *NPM1*-negative test result were excluded from the data analysis without repeated testing. A Deming regression analysis was performed on the log-transformed (LT) quantitative measurements of *NPM1* mutant RNA relative to reference gene *ABL1* RNA. Additionally, a Deming regression analysis was performed on the percentage ratio [*NPM1* mutant RNA divided by *ABL1* RNA] between the quantitative results from the Xpert NPM1 mutation assay and the Ipsogen assay. Bland–Altman plots were used to evaluate bias between the test quantitation outputs for the Xpert and Ipsogen assay results. 

## Figures and Tables

**Table 1:** Results of Xpert NPM1 mutation assay and comparator method tests (percent ratio).

		Percent Ratio (NPM1 to ABL1)		Mutation A, B, or D
Specimen	Dilution Scheme	Ipsogen NPM1	Xpert NPM1	
1	1:1	3.3096	3.3200	A
2	1:1	1.5609	1.1830	A
3	1:1	2.3019	2.0450	B
4	1:1	3.8340	3.9100	A
5	1:1	3.2714	3.6270	A
6	1:1	0.3759	0.6725	A
7	1:1	0.9248	0.9957	A
8	1:1	2.3121	3.2980	D
11	1:10	0.2006	0.6588	B
12	1:10	0.0040	0.0545	B
13	1:10	0.1708	0.2855	A
14	1:10	0.2191	0.3302	A
15	1:10	0.2292	0.2868	A
16	1:10	0.4061	0.9623	D
17	1:10	0.1032	0.1472	A
18	1:10	0.0473	0.0826	A
19	1:10	0.0238	0.0300	A
21	1:100	0.0173	0.0358	A
22	1:100	0.0249	0.0401	A
23	1:100	0.0098	0.0240	B
24	1:100	0.0284	0.0425	A
25	1:100	0.0318	0.0589	A
26	1:100	0.0485	0.1274	D
27	1:100	0.0147	0.0259	A
28	1:100	0.0248	0.0371	A
29	1:100	0.0355	0.0397	A
30	1:100	0.0251	0.0336	A
31	1:1000	0.0007	0.0014	A
32	1:1000	0.0025	0.0030	A
33	1:1000	0.0016	0.0036	A
34	1:1000	0.0030	0.0049	A
35	1:1000	0.0012	0.0022	A
36	1:1000	0.0003	0.0015	B
37	1:1000	0.0036	0.0037	A
38	1:1000	0.0033	0.0049	A

## Data Availability

Data is contained within the article.

## References

[1] Döhner H., Weisdorf D.J., Bloomfield C.D. (2015). Acute Myeloid Leukemia. N. Engl. J. Med..

[2] Acute Myeloid Leukemia—Cancer Stat Facts. National Cancer Institute—Surveillance, Epidemiology, and End Results Program. https://seer.cancer.gov/statfacts/html/amyl.html.

[3] Kunchala P., Kuravi S., Jensen R., McGuirk J., Balusu R. (2018). When the good go bad: Mutant NPM1 in acute myeloid leukemia. Blood Rev..

[4] Coleman W.B., Coleman W.B., Tsongalis G.J. (2016). Molecular Testing in Acute Myeloid Leukemia. Diagnostic Molecular Pathology—A Guide to Applied Molecular Testing.

[5] Pollyea D.A., Altman J.K., Assi R., Bixby D., Fathi A.T., Foran J.M., Gojo I., Hall A.C., Jonas B.A., Kishtagari A. (2023). Acute myeloid leukemia, version 3.2023, NCCN clinical practice guidelines in oncology. J. Natl. Compr. Cancer Netw..

[6] Forghieri F., Comoli P., Marasca R., Potenza L., Luppi M. (2018). Minimal/Measurable Residual Disease Monitoring in NPM1-Mutated Acute Myeloid Leukemia: A Clinical Viewpoint and Perspectives. Int. J. Mol. Sci..

[7] Alpermann T., Schnittger S., Eder C., Dicker F., Meggendorfer M., Kern W., Schmid C., Aul C., Staib P., Wendtner C.-M. (2016). Molecular subtypes of NPM1 mutations have different clinical profiles, specific patterns of accompanying molecular mutations and varying outcomes in intermediate risk acute myeloid leukemia. Haematologica.

[8] Lane D.M. (2019). Measures of Variability. Online Statistics Education: An Interactive Multimedia Course of Study.

[9] Döhner H., Wei A.H., Appelbaum F.R., Craddock C., DiNardo C.D., Dombret H., Ebert B.L., Fenaux P., Godley L.A., Hasserjian R.P. (2022). Diagnosis and management of AML in adults: 2022 recommendations from an international expert panel on behalf of the ELN. Blood.

[10] Freeman S.D., Hourigan C.S. (2019). MRD evaluation of AML in clinical practice: Are we there yet?. Hematol. Am. Soc. Hematol. Educ. Program.

[11] Xpert NPM1 Mutation RUO IFU ENGLISH 302-5822 Rev. B. https://www.cepheid.com.

[12] Gorello P., Cazzaniga G., Alberti F., Dell’Oro M.G., Gottardi E., Specchia G., Roti G., Rosati R., Martelli M.F., Diverio D. (2006). Quantitative assessment of minimal residual disease in acute myeloid leukemia carrying nucleophosmin (NPM1) gene mutations. Leukemia.

